# Dosimetric evaluation of radiation dose rate effect in respiratory gated intensity modulated radiation therapy†

**DOI:** 10.2349/biij.8.1.e5

**Published:** 2012-01-01

**Authors:** C Khamfongkhruea, C Tannanonta, S Thongsawad

**Affiliations:** Radiation Oncology Unit, Chulabhorn Hospital, Bangkok, Thailand.

**Keywords:** Fine needle aspiration (FNA), pneumothorax, lung lesion, biopsy, CT

## Abstract

**Purpose:**

To investigate the dosimetric accuracy of the sliding window gated IMRT compared with the static treatment, using varying dose rates.

**Materials and methods:**

This study measured changes in output and diode array response with changing dose rate, verified the precision of the motion table, and measured changes in dose distribution accuracy with film and diodes at two depths with changing dose rate. During 4DCT (4 Dimensional Computed Tomography), the patient’s respiratory signals and target motion were recorded and imported to the XY4D simulation table of SUN NUCLEAR Corporation to simulate the patient’s respiration and tumour motion. A single field of each sliding window IMRT plan with 30º wedge and one for lung cancer were used in this study. Three irradiating conditions, static and moving target with and without gating, were applied to both plans.

**Results:**

The standard deviations of output, with the dose rates changing from 300–600 MU/min, were 0.065 cGy and 0.169 cGy for the ionisation chamber and diode, respectively. The verification of the motion table shows very good precision with 9.98 ± 0.02 cm (true value = 10.0 cm). The measurements by MapCheck show the gamma index of the planned absolute dose distribution in static and moving targets with gating, resulting in more than 96% passing for all dose rates. The absolute dose distribution measured by film for the static target was agreeable with the value of moving target with gating.

**Conclusion:**

The sliding window gated IMRT technique is able to deliver an accurate dose to a moving target with the dose rate of 300–600 MU/min that is suitable for clinical treatment.

## INTRODUCTION

Intensity modulated radiotherapy (IMRT) utilises small beamlets of ionising radiation to provide a high radiation dose to a target while at the same time reducing the dose to normal organs [1]. However, dose uncertainties of IMRT technique occur when the treated target volume moves outside the beam aperture and the normal organ near the target can receive a high dose if it moves into the field [2]. It is well known that target motion due to respiration is a significant and challenging problem in radiation therapy [3, 4]. Several approaches have been developed to manage the effects of respiratory motion in radiation therapy. One of the approaches is to use a respiratory gating system. However, the disadvantage of this technique is that it is time-consuming. Thus the use of high dose rate delivery has been applied with the gated IMRT to overcome this problem.

There have been many studies on the interplay effects of moving target irradiated with gated IMRT. Chen *et al*. [5] studied the dosimetric effects caused by the respiratory motion during IMRT by using Kodak EDR2 films. They concluded that, without the gating system, the dose distribution of the stationary phantom was different from the moving one. The limited residual motion to less than 0.5 cm was critical for moving target treatments. The gating window size and delivery method of gated IMRT were considered by Hugo *et al*. [6]. Their results show that the gating window size affected the dosimetric error which was reduced by reducing window size. Duan *et al*. [7] studied the dosimetric effect of respiration-gated beam with IMRT delivery. Their results suggested that low dose rate can reduce the effect of delay and catch-up cycle. This effect is the phenomenon whereby beam hold-offs in gated delivery allow the lagging leaves to catch up with the delivered monitor units each time that the beam is interrupted. A good balance between the rapid dose delivery and delivery accuracy should be determined, which agrees with the results of Ehler *et al*. [1]. The MU accuracy of gated step-and-shoot IMRT was reported by Cheong *et al*. [8]. The gated IMRT delivery demonstrated an MU accuracy that was equivalent to the ungated IMRT and there was good agreement between the delivered MUs with gating and the planed MU within + 0.5 MU regardless of dose rate and duty cycle. Lin *et al*. [2] determined the effect of radiation dose rate with moving target and the gated treatment using step-and-shoot IMRT delivery. As a result, the IMRT step-and-shoot delivery with gating for 1000 MU/min did not show much difference from 500 MU/min. However, the high dose rate gated step-and-shoot IMRT was dosimetrically accurate, shortened the delivery time, and was safe to use clinically.

Although the sliding window IMRT delivery errors may be higher than the step-and-shoot technique [6], but the treatment time is reduced. The purpose of this study was to investigate the accuracy of the sliding window gated IMRT comparing with the static treatment, using varying dose rates.

## MATERIALS AND METHODS

### Dose rate dependence

#### Radiation output

To verify the variation of radiation output with dose rate, a cylindrical ionisation chamber (FC65-P of IBA Dosimetry) with Dose 1 electrometer (IBA Dosimetry) was used. The measurement was made in a solid water phantom at 10 cm depth, 10×10 cm^2^ filed size for 100 MU of 6 MV photon beams using the dose rates of 300, 400, 500, and 600 MU/min. The output value for each rate was obtained from ten measurements.

#### 2D diode array response

A MapCheck 2D diode array of SUN NUCLEAR Corporation was used for dose measurement. It was calibrated for both sensitivity and absolute dose [9] and the dose rate dependence was checked by measuring the dose with the same irradiation conditions of A.1.

### Phantom simulation

#### Patient’s data analysis

**Figure 1:**
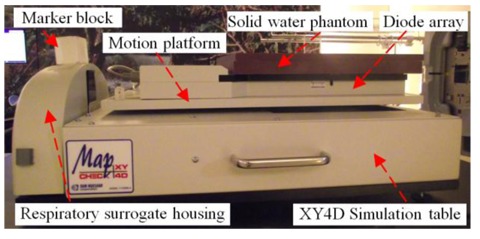
The experimental setup of diode array with XY4D simulation table.

The data of a lung cancer patient with large tumour motion (2 cm longitudinal direction) was selected for this study. The patient’s respiratory signal and tumour motion data from 4DCT simulation (Brilliance CT Big Bore, Philips) with Real-time Position Management™ (RPM) system (Varian Medical System Palo Alto, CA) version 1.7.5 were analyzed. Then the respiratory signal for the selected patient (2 cm amplitude and 5.5 sec/cycle respiratory rate) was imported to the XY 4D motion simulation table as shown in Figure 1. The motion table has a respiratory surrogate housing which can move in a vertical direction to simulate respiratory sinusoidal wave and a motion platform which is capable of simultaneous two-dimensional motions (longitudinal and lateral directions) with stepper motors. The surrogate housing and motion platform are controlled by a personal computer with MapCheck software. In this study, the motion table simulated the tumour movement in a longitudinal direction which is perpendicular with the axis of sliding MLC movement.

#### Accuracy and reproducibility of XY4D motion simulation table

The motion table was controlled to stop at 50% respiratory phase (middle value of the gating window normally used to treat the patient but the CT reconstruction cannot be done for the middle value of 55% phase). A lead wire marker was placed on the motion platform and another one on the stationary simulation table with 10 cm separation. The 4DCT scan of the motion table and wires was performed with gating, using the previously mentioned patient respiratory signal. This 4DCT scan was repeated three times.

### Measurement

A Varian Trilogy linear accelerator with 120 leaves Millennium MLC was used to deliver the treatment planning dose. An Eclipse treatment planning system version 8.1.20 (Varian Medical System Palo Alto, CA) was used to generate two IMRT plans with sliding window of single field. One plan was generated to simulate the fluence for 30º wedge and another one for a patient with lung cancer. Three irradiating conditions, one static and two moving targets with and without gating, were applied to both plans. For the moving target with gating, the gating window of 40%–70%, respiratory cycle (to have equal to, or less than, 0.2 cm residual motion) was set. A Gafchromic EBT film was placed on the 2D diode array on the simulation table with 3 cm of solid water phantom above the film to measure the doses at 3 cm and 5 cm depths by the film and 2D array, respectively. Four steps of dose rate 300, 400, 500, and 600 MU/min were applied for irradiation of each plan.

The dynamic log files for Millennium MLC on 4D Integrated Treatment Console (4DITC) workstation were also generated during the treatment to evaluate the leaf position difference between the treatment and plan.

The MapCheck software was used to analyze the 2D dose distribution measured by the 2D diode array and Gafchromic film. Then the measured and planned data were compared using the gamma index (3% and 3 mm).

A Leaf Error RMS (Root Mean Square) value of DynaLog files was analyzed using Varian DynaLog file viewer software. The value was calculated by the equation:

$$Leaf\;Error\;RMS=\sqrt{\frac{\sum_{t=1}^{n}{\left(LeafPlanPos_{t}-LeaftActualPos_{t}\right)}^{2}}{n}}$$

where t = data sample index, n = total number of samples.

## RESULTS AND DISCUSSION

### Dose rate dependence

The standard deviation values of the measurements with the dose rates of 300–600 MU/min were 0.065 cGy and 0.169 cGy for the ionisation chamber and diode, respectively.

**Figure 2:**
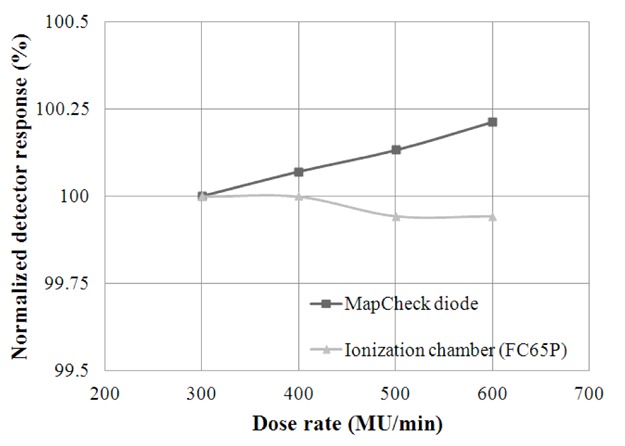
Dose rate dependence of the ionisation chamber and central diode of the MapCheck.

As shown in Figure 2, the output of the machine with 6 MV measured by the chamber was quite constant for this range of dose rates with the maximum variation of − 0.06% for dose rate 600 MU/min (normalised with the dose rate of 300 MU/min).

The response of the diode increased with rising dose rate value with the maximum variation of 0.21%, which is satisfactory and comparable with the value of 0.35% from Le´tourneau *et al*. [9].

### Phantom simulation

**Figure 3:**
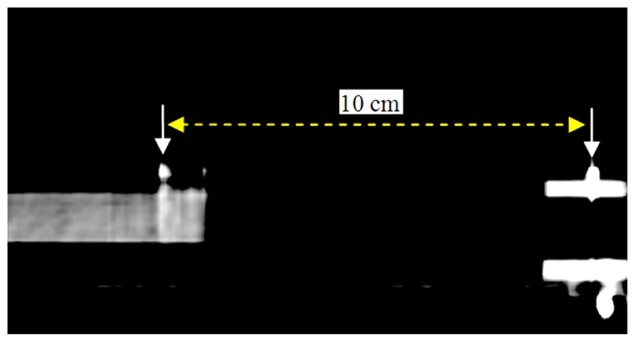
The sagittal CT image of moving phantom with 50% phase showing the distance between the lead wire markers: one on motion platform (left arrow) and another one on stationary platform (right arrow).

Figure 3 shows a 4DCT sample with the separation of two markers. The average value of the separation was very close to the true value with 9.98 ± 0.02 cm, so that the motion table accuracy is acceptable.

### Measurement

Table 1 illustrates the comparison of the measured (by diode) and planned absolute dose distribution using the gamma index values (3%, 3 mm). The percentage of passing was more than 96% for static and moving target with gating, and less than 85.7% for moving target without gating in both IMRT plans. However, the dose rate dependence of MapCheck diode in the operating range should be considered. The percentage of passing for static lung plan was lower than the wedge field because of the complexity of intensity fluence map. The lung plan shows worse results with lower dose rate, which may be because of the longer treatment time (higher chance of tumour moving outside of the treatment field).

**Table 1:** The gamma index of comparison of IMRT dose distribution between Mapcheck diode and planned dose distribution for various dose rates.

**Dose rate (MU/min)**	**30° Wedge plan**			**Lung plan**		
	**Static**	**Moving +no gating**	**Moving +gating**	**Static**	**Moving +no gating**	**Moving +gating**
300	99.9 ± 0.1	82.5 ± 0.7	99.7 ± 0.1	97.0 ± 0.2	46.2 ± 15.8	96.2 ± 0.3
400	100.0 ± 0.1	81.8 ± 0.6	99.7 ± 0.1	97.2 ± 0.2	45.9 ± 13.6	96.0 ± 0.6
500	100.0 ± 0.1	84.1 ± 0.1	99.7 ± 0.0	97.2 ± 0.2	61.6 ± 3.5	96.2 ± 0.3
600	100.0 ± 0.1	85.7 ± 1.0	99.7 ± 0.1	97.2 ± 0.2	53.4 ± 6.1	96.1 ± 0.5

**Figure 4:**
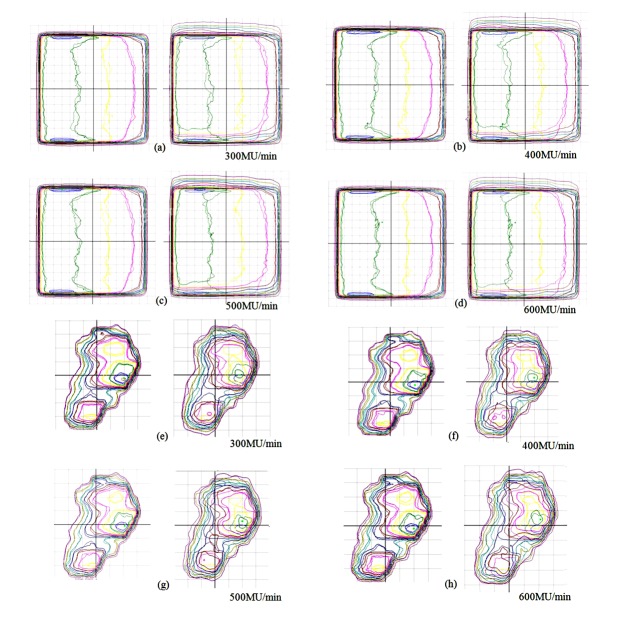
The dose distribution of film, comparing between static and moving target with gating (left) and without gating (right) for 30º wedge IMRT field (a-d) and lung IMRT plan (e – h). (Dashed lines for static and solid lines for moving target with and without gating).

The dose distribution measured by film for the moving target with both gating and without gating (Figure 4) was compared with the value of the static target (gold standard). As shown in Table 2, the percentage of agreement evaluated by using gamma index (3%, 3 mm) was about 100% for both plans with gating for all dose rates, but the value was lower without gating and even lower with more complex plan of lung (52.5%–58.7%) than 30º wedge (76.5%–87.7%).

**Table 2:** The percentage of agreement of the measured dose distribution using film for moving target compared with the value of static target for various dose rates.

**Dose rate (MU/min)**	**30° Wedge plan**		**Lung plan**	
	**Moving +no gating**	**Moving +gating**	**Moving +no gating**	**Moving +gating**
300	76.5 ± 2.1	100 ± 0	58.7 ± 0.3	99.9 ± 0.1
400	87.7 ± 0.2	100 ± 0	52.5 ± 0.8	100 ± 0.1
500	83.5 ± 0.7	100 ± 0	54.9 ± 0.1	99.9 ± 0.1
600	84.2 ± 1.1	100 ± 0	54.6 ± 0.1	100 ± 0.1

Table 3 shows that the leaf position error was increased with rising dose rate for every technique but the error of the gating was not significantly different from static value with p-value more than 0.114 for both IMRT treatments. This result agrees with the report of Ahunbay *et al*. [10].

**Table 3:** The maximum leaf error RMS in cm of DynaLog files.

**Dose rate (MU/min)**	**30° Wedge plan**				**Lung plan**			
	**Blank A**		**Blank B**		**Blank A**		**Blank B**	
	**No gating**	**Gating**	**No gating**	**Gating**	**No gating**	**Gating**	**No gating**	**Gating**
300	0.026	0.026	0.021	0.021	0.028	0.027	0.035	0.035
400	0.030	0.031	0.031	0.030	0.035	0.035	0.046	0.045
500	0.035	0.035	0.036	0.035	0.043	0.043	0.055	0.054
600	0.038	0.038	0.040	0.039	0.051	0.050	0.064	0.063

## CONCLUSIONS

From the measurements using the diode array, the percentage of passing for dose distribution of dynamic IMRT plan with gating is acceptable with ≥ 96% for the dose rate of 300–600 MU/min. The passing value will be decreased if the fluence map is more complicated. The dose distribution (measured with EBT film) is the same for static and moving targets with gating. The leaf position error of the gating treatment is not significantly different from the static target treatment but the error is increased with rising dose rate up to 600 MU/min. For the IMRT treatment of the moving target, gating should be utilised. The multileaf collimator positioning accuracy is affected by the dose rate but not by the gating. However, there were very few leaf position errors with the maximum dose rate, and the dose distribution is still the same as the static target treatment. The high dose rate of up to 600 MU/min can be used with gating to decrease the dose uncertainties from target movement. Furthermore, for dosimetric accuracy, every patient-specific QA procedure with simulation of phantom movement should be performed.

## References

[1] Ehler ED, Tomé WA (2009). Step and shoot IMRT to mobile targets and techniques to mitigate the interplay effect. Phys Med Biol.

[2] Lin T, Chen Y, Hossain M, Li J, Ma CM (2008). Dosimetric investigation of high dose rate, gated IMRT. Med Phys.

[3] Keall PJ, Mageras GS, Balter JM, Emery RS, Forster KM, Jiang SB, Kapatoes JM, Low DA, Murphy MJ, Murray BR, Ramsey CR, Van Herk MB, Vedam SS, Wong JW, Yorke E (2006). The management of respiratory motion in radiation oncology report of AAPM Task Group 76. Med Phys.

[4] Wong KH, Dieterich S, Tang J, Cleary K (2007). Quantitative measurement of CyberKnife robotic arm steering. Technol Cancer Res Treat.

[5] Chen H, Wu A, Brandner ED, Heron DE, Huq MS, Yue NJ, Chen WC (2009). Dosimetric evaluations of the interplay effect in respiratory-gated intensity-modulated radiation therapy. Med Phys.

[6] Hugo GD, Agazaryan N, Solberg TD (2002). An evaluation of gating window size, delivery method, and composite field dosimetry of respiratory-gated IMRT. Med Phys.

[7] Duan J, Shen S, Fiveash JB, Brezovich IA, Popple RA, Pareek PN (2003). Dosimetric effect of respiration-gated beam on IMRT delivery. Med Phys.

[8] Cheong KH, Kang SK, Lee M, Kim SS, Park S, Hwang TJ, Kim KJ, Oh do H, Bae H, Suh TS (2010). Evaluation of delivered monitor unit accuracy of gated step-and-shoot IMRT using a two-dimensional detector array. Med Phys.

[9] Létourneau D, Gulam M, Yan D, Oldham M, Wong JW (2004). Evaluation of a 2D diode array for IMRT quality assurance. Radiother Oncol.

[10] Ahunbay E, Li XA (2007). Investigation of the reliability, accuracy, and efficiency of gated IMRT delivery with a commercial linear accelerator. Med Phys.

